# User experience study to evaluate a clinical decision support system prototype supporting continuous kidney replacement therapy in a simulated ICU environment

**DOI:** 10.1186/s12911-025-03165-7

**Published:** 2025-09-10

**Authors:** L-M. Kunz, M. Metzger, C. Schaefer, R. Pohlmeier, J. Petrovic Vorkapic, Michael Nosch

**Affiliations:** 1 Fresenius Medical Care Deutschland GmbH, Bad Homburg, Germany; 2 Information Technology Marienhospital, Bottrop, Germany; 3 Intensive Care Medicine Marienhospital, Bottrop, Germany; 4 Emergency Department, Helios Spital, Überlingen, Germany

**Keywords:** User experience, Acute kidney injury, Clinical decision support system, Continuous kidney replacement therapy, Digital health technologies

## Abstract

**Background:**

The increasing amount of data routinely collected on ICUs poses a challenge for clinicians which is aggravated with data-heavy therapies like Continuous Kidney Replacement Therapy (CKRT). We developed the CKRT Supporting Software Prototype (CKRT-SSP), a clinical decision support system for use before, during and after CKRT. The aim of this user experience (UX) study was to prospectively evaluate CKRT–SSP in terms of usability, user experience, and workload in a simulated ICU setting.

**Methods:**

We simulated CKRT treatments in a fully equipped single patient room in the ICU and evaluated CKRT-SSP using validated questionnaires: System Usability Scale (SUS) and User Experience Questionnaire (UEQ). Furthermore, a modified NASA-TLX (task load index) compared the workload before and after using CKRT-SSP. Twelve clinicians and nurses participated in this study.

**Results:**

The SUS reached a median value of 87.5 for CKRT-SSP, reflecting excellent usability. In the UEQ, CKRT-SSP scored clearly positive in the attractiveness dimension and the three task-related dimensions of clarity, efficiency, and dependability (95% CI fully > 0.8). For the two non-task-related dimensions, stimulation and novelty, there was a positive trend (mean > 0.8, lower limit of 95% CI < 0.8). The modified NASA-TLX suggests a trend to less total workload with CKRT-SSP which mainly is attributable to less physical demand and less effort.

**Conclusion:**

CKRT-SSP is a promising tool for improving the workload in ICUs and the specific application of CKRT. We obtained valuable insights for further user-centric development.

**Supplementary information:**

The online version contains supplementary material available at 10.1186/s12911-025-03165-7.

## Background

Hospitals are now using and relying on a wide range of software, particularly in a demanding and costly setting like the intensive care unit (ICU). There is an increasing demand for interoperable systems, integrated monitoring systems, and comprehensive support software to facilitate data utilization and precision medicine in these high-workload environments [1, 2].

An example of an established software system is the “Patient Data Management System” (PDMS), a computer-based system that combines patient monitoring and the Hospital Information System (HIS) to support the processing of medical data in ICUs. PDMSs main task is to streamline documentation and work processes of health care professionals (HCP) by automatically collecting and transferring data from medical devices such as monitors, ventilators, renal replacement devices, laboratory systems and many others [3].

With a PDMS the documentation largely runs automatically which saves time and resources (paperless working), however the amount of data a HCP needs to handle when treating critically ill patients at the ICU, is enormous. A study from a Canadian group reported that the care of critically ill patients generates a median of 1348 individual data points per day [4]. A contributor to the data load is acute kidney injury (AKI), which affects a subgroup of critically ill patients and often requires continuous kidney replacement therapy (CKRT). The proportion of ICU AKI cases requiring CKRT ranges from 5 to 26% [5, 6]. CKRT is a data-heavy intervention, requiring frequent adjustments of therapy settings such as blood and dialysate flows, fluid removal targets, and anticoagulation dosing. Additionally, it involves continuous tracking of therapy delivery, alarms, patient responses, and fluid balance across 24-hour cycles. This creates a constant stream of interdependent data points that must be interpreted in real time [7]. Due to such abundance of data, clinicians face constraints in fully utilizing the entire spectrum for decision-making at the point of care [4].

Handling this amount of various data requires cognitive resources. Such high cognitive workload can generate cognitive overload which leads to higher error rates due to interruptions of tasks. In an ICU this can cause life threatening situations [8]. It has been suggested that clinical decision support systems (CDSS), which digest data for therapeutic decision making and handling of various therapy systems, have multiple benefits including a potential to reduce workload and mitigate the risk of cognitive overload [2, 8–10]. Despite the benefits, CDSS implementation remains challenging, due to multiple factors, including clinician acceptance which may be driven by user experience, technical integration, workflow alignment, and organizational support [11]. Analyzing impact on the workflow and incorporating user experience into the development process is important to enhance clinician acceptance and optimize CDSS adoption [11].

We have developed a CDSS prototype specifically dedicated to support HCPs before, during and after Continuous Kidney Replacement Therapy (CKRT) of AKI in ICUs: CKRT Supporting Software Prototype (CKRT-SSP). The aim of this user experience (UX) study was to evaluate the impact of CKRT–SSP on usability, user experience and workload in a simulated ICU environment.

## Methods

### Study design

To investigate the benefit of the CKRT-SSP the following hypotheses were tested at the Marienhospital Bottrop Germany in a non-clinical UX-study.

- H1: CKRT-SSP usage is perceived as effective, efficient, and satisfying.
- H2: CKRT-SSP is perceived as attractive, perspicuous, efficient, dependable, stimulating, and novel.
- H3: adding CKRT-SSP to current CKRT prescription and monitoring (without software system) is expected to lead to a workload reduction.

**Fig. 1:**
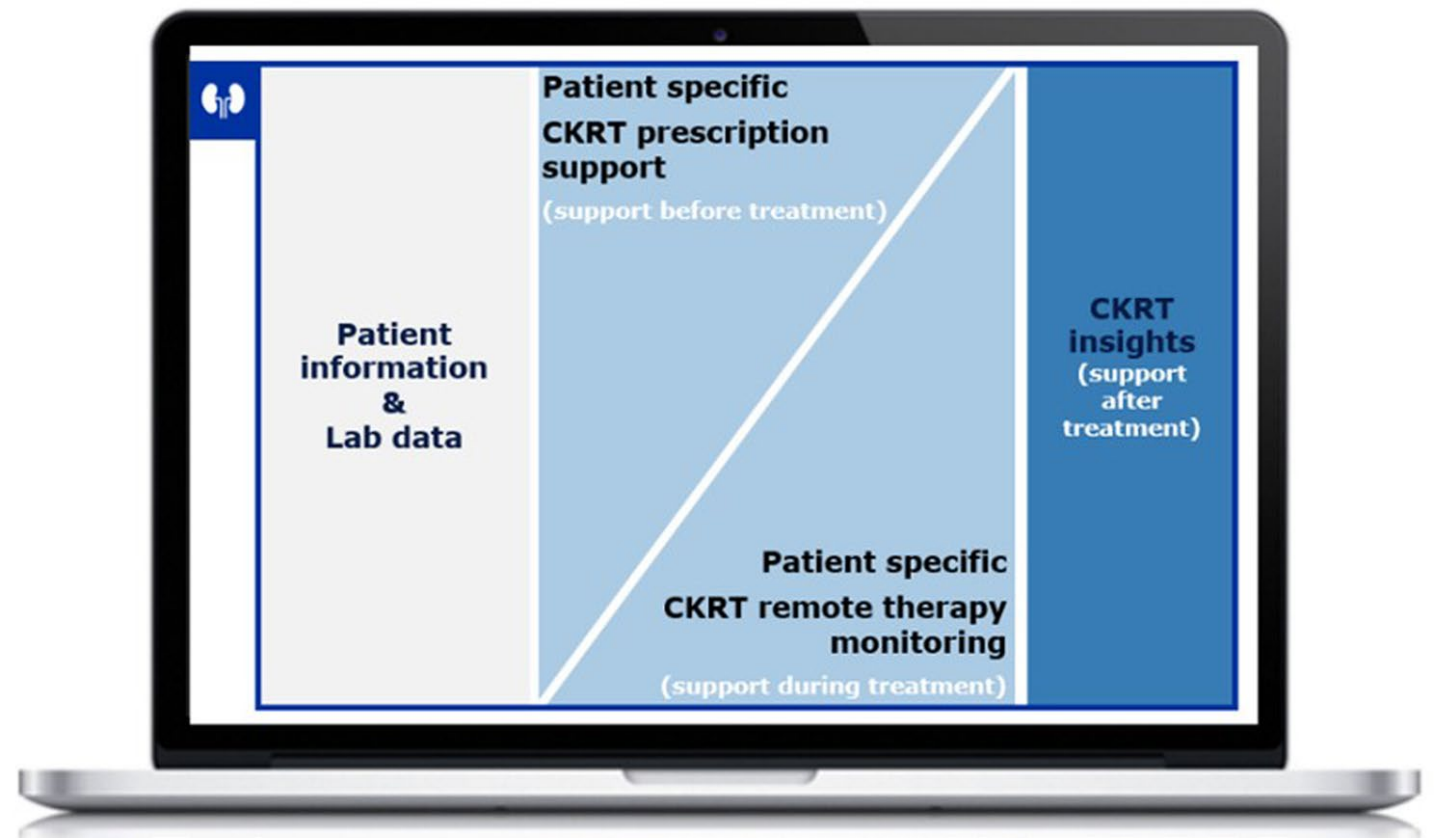
Description of CKRT-SSP – left: relevant patient and lab data; middle: patient centric CKRT view; right: retrospective CKRT quality measures

The CKRT-SSP is designed for supporting HCPs in the ICU with the management of critically ill patients with acute kidney injury (AKI) and need for CKRT. It is a “stand alone” web-based software which is installed and currently accessible on a desktop computer, e.g., at the central point in the ICU, at the patient bed side, in the “physician´s room” or in the operating room. It leverages several data points from direct connection to the CKRT device, blood gas analyzer and PDMS. By consolidating patient, blood sample, and CKRT data, the CKRT-SSP offers clinical staff before treatment prescription support, during treatment remote CKRT monitoring, and after treatment retrospective insights and quality metrics (Fig. 1). This integrated approach aims to minimize guesswork in individual prescriptions, provide a comprehensive, single-view interface that reduces the need for mentally processing multiple, relevant data points, and enhance clinical decision-making and efficiency. The CKRT-SSP was developed following the Human-Centered Design (HCD) process, a user-centric methodology based on cognitive science, systems thinking, and design theory, which emphasizes the integration of end-user needs, preferences, limitations, and behaviors throughout the design and development phases [12].

### Study participants

Given a small hospital setting, a total of 12 HCPs were recruited from the ICU at Marienhospital Bottrop to partake in this UX-study. Scheduling participation had to be coordinated around ongoing ICU responsibilities to avoid disrupting patient care. In Phase 1, 8 participants were involved, evenly split between intensive care nurses and physicians with a diverse range of professional experience and seniority. The nurses had between 8 and 27 years of experience (mean: 16.75 years), while the physicians had 5 to 20 years (mean: 11.5 years). In Phase 2, also 8 participants, including 2 physicians and 6 nurses, were involved, four of the participants (2 nurses and 2 physicians) of Phase 2 had previously participated in Phase 1. Nursing experience ranged from 4 to 35 years (mean: 13.5 years), and physicians from 5 and 20 years of experience (mean: 12.5 years). Informed written consent was obtained from all participants, and they were informed of the study’s purpose and procedures before their involvement.

### Study procedure

The evaluation phase included a prospective “before-and-after” study. In the initial phase of the study phase “before”, an exploration of the existing clinical workflow and processes with regards to the prescription and monitoring of CKRT was undertaken through self-created qualitative baseline interview employing open-ended questions based on a structured questionnaire (see additional file 1) and a quantitative questionnaire NASA-TLX [13].

**Fig. 2:**
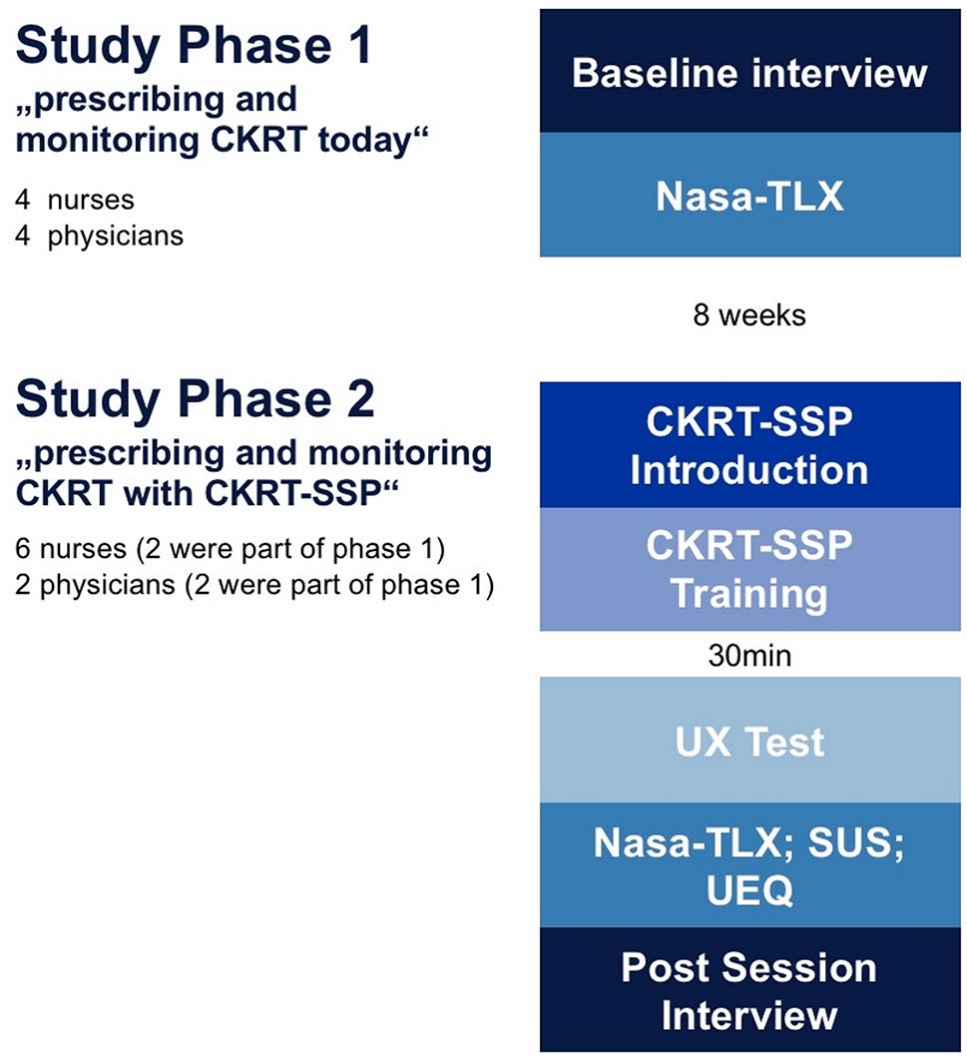
CKRT-SSP UX study overview

As part of the subsequent phase 2 “after”, CKRT-SSP was installed in the real-world IT environment of the ICU at Marienhospital Bottrop. Eight participants received 20 min of structured training before the testing (see additional file 2). In the testing phase, the participants were asked to solve tasks using CKRT-SSP. These tests took place in a fully equipped, unoccupied single patient room in the ICU with simulated CKRT treatments: a CKRT device was set up and brought into circulation to resemble the real ICU setting, but no patient was directly involved. The work has been carried out independently on the prototype and included tasks in the areas of “CKRT prescription” before a CKRT therapy, “treatment monitoring” during a CKRT therapy, and “treatment analysis” after completed CKRT therapies. The tasks included but were not limited to: creating a new patient record, assigning a bed location, and developing a new CKRT prescription for a simulated patient using dummy blood gas analysis data to monitor an ongoing CKRT. This was followed by an interview with open post-testing questions (see additional file 3) and the quantitative NASA-TLX questionnaires (Fig. 2). The study method met ISO 9241–11 standards for usability, covering effectiveness, efficiency, and user satisfaction.

### Measurement instruments - quantitative questionnaires

- SUS- System Usability Scale [14] – Evaluates perceived usability (H1) using a validated 10-question, 5-point Likert scale questionnaire, providing a score from 0 to 100. Bangor et al. standardized SUS interpretation with adjective ratings and descriptions of acceptable scores [15].
- UEQ [16] – Evaluates the use and attractiveness (H2) measuring both classical usability (efficiency, clarity [originally termed perspicuity], dependability) and user experience (originality, stimulation) aspects. It consists of 26 opposing word pairs grouped into six scales (“Attractiveness”, “Clarity”, “Efficiency”, “Dependability”, “Stimulation” and “Novelty”) rated on a 7-point Likert scale from −3 (negative) to + 3 (positive), with scores interpreted as negative ( < −0.8), neutral (−0.8 to 0.8), or positive ( > 0.8) [17, 18]. Task-related dimensions (clarity, efficiency, dependability) reflect how easily and effectively users can interact with the system in relation to their tasks. Non-task-related dimensions (stimulation, novelty) relate to the emotional and aesthetic aspects of the user experience, such as how engaging or innovative the system feels.
- NASA-TLX [13] – Evaluates of the perceived workload (H3 across six subscales: Mental Demands, Physical Demands, Temporal Demands, Performance, Effort, and Frustration. Originally designed for aircraft leadership tasks, it uses a 21-step scale from 0 to 20, with higher scores indicating higher workload. These ratings for each dimension are then combined to the task load index [13]. In this study, only the first part of NASA-TLX, the rating scale, was used, omitting the pair-wise weighting [19]. It was applied before and after the study phases. Results are completely displayed in graphical format including indicating paired and unpaired data points.

## Results

### SUS results

**Fig. 3:**
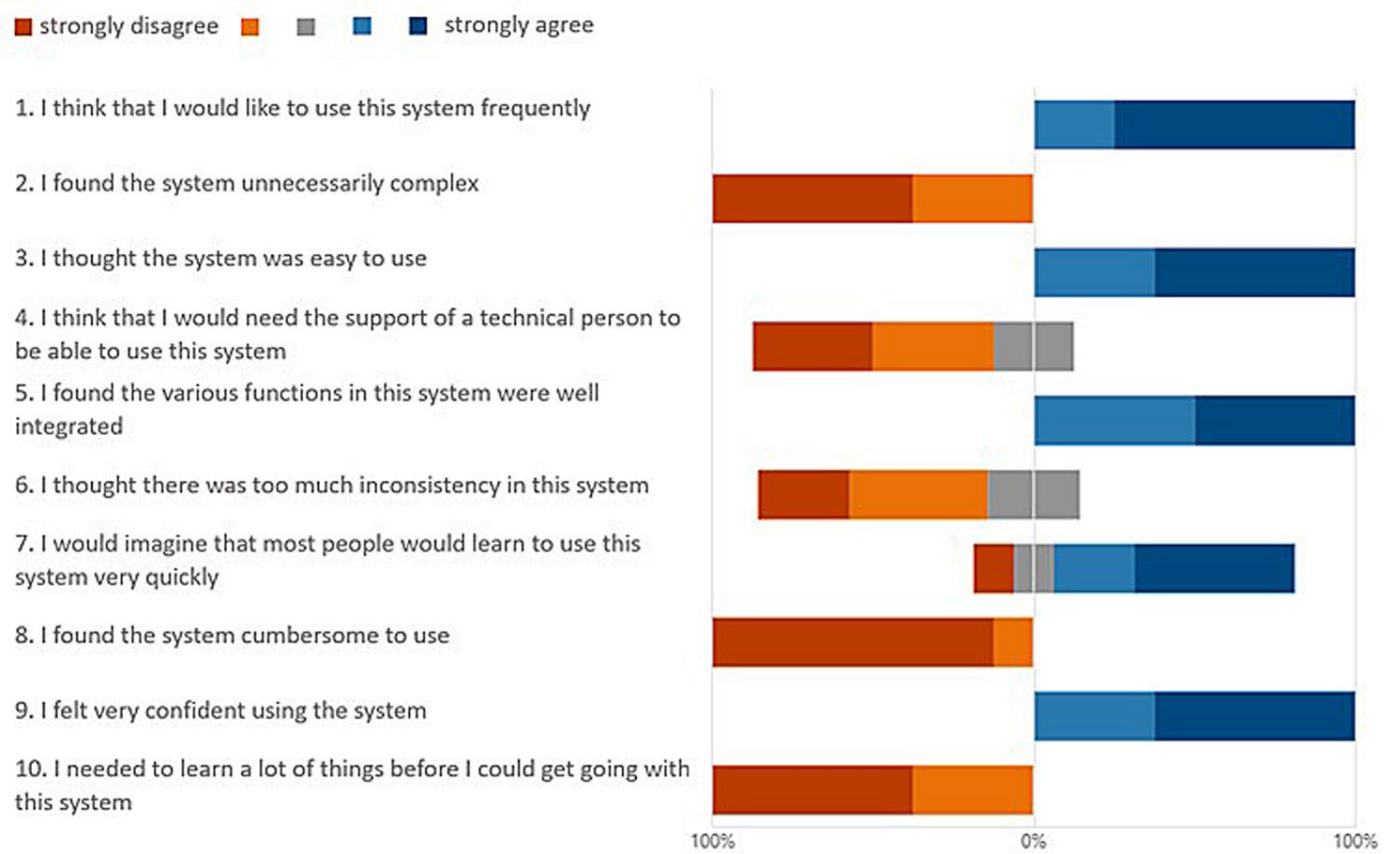
Answer distribution of the SUS - System usability scale for CKRT-SSP

The SUS of the CKRT-SSP resulted in a mean of 87.5 (n: 8; SD: 8.39). Using the assessments by Bangor et al., this value is within the acceptable range (70 to 100) and an adjective rating of “excellent” is appropriate, as applied to studies with mean SUS results of 85.58 (SD: 9.473) [15]. Fig. 3 illustrates the detailed distribution of specific answers on the SUS for CKRT-SSP.

### UEQ results

**Fig. 4:**
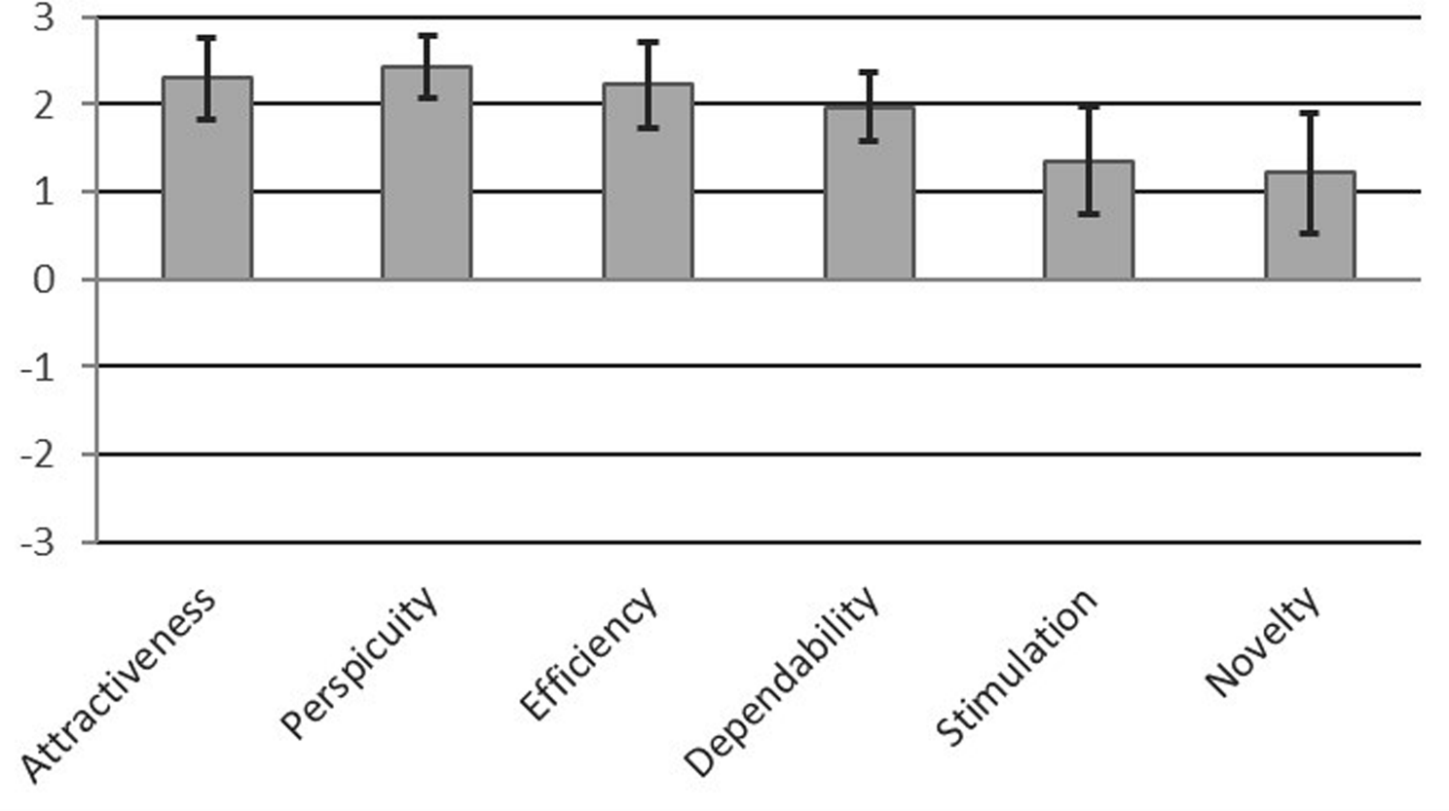
Graphical visualization of the scale means of the UEQ scales

UEQ results showed that CKRT-SSP scored positive in the dimension attractiveness and the three task related dimensions clarity, efficiency, and dependability (both ends of the 95% CI > 0.8, briefly 95% CI fully > 0.8). For the two non-task related dimensions stimulation and novelty there was a positive trend (mean > 0.8, while lower limit of 95% CI < 0.8). See also Table 1 and Fig. 4.

**Table 1:** Confidence intervals and the scale means of the UEQ scales

Scale	Mean	Std. Dev.	N	95% confidence interval	
Attractiveness	2.29	0.68	8	1.82	2.76
Clarity	2.44	0.51	8	2.08	2.79
Efficiency	2.22	0.70	8	1.73	2.70
Dependability	1.97	0.57	8	1.57	2.37
Stimulation	1.35	0.87	8	0.74	1.96
Novelty	1.22	0.99	8	0.53	1.91

### NASA-TLX results

**Fig. 5:**
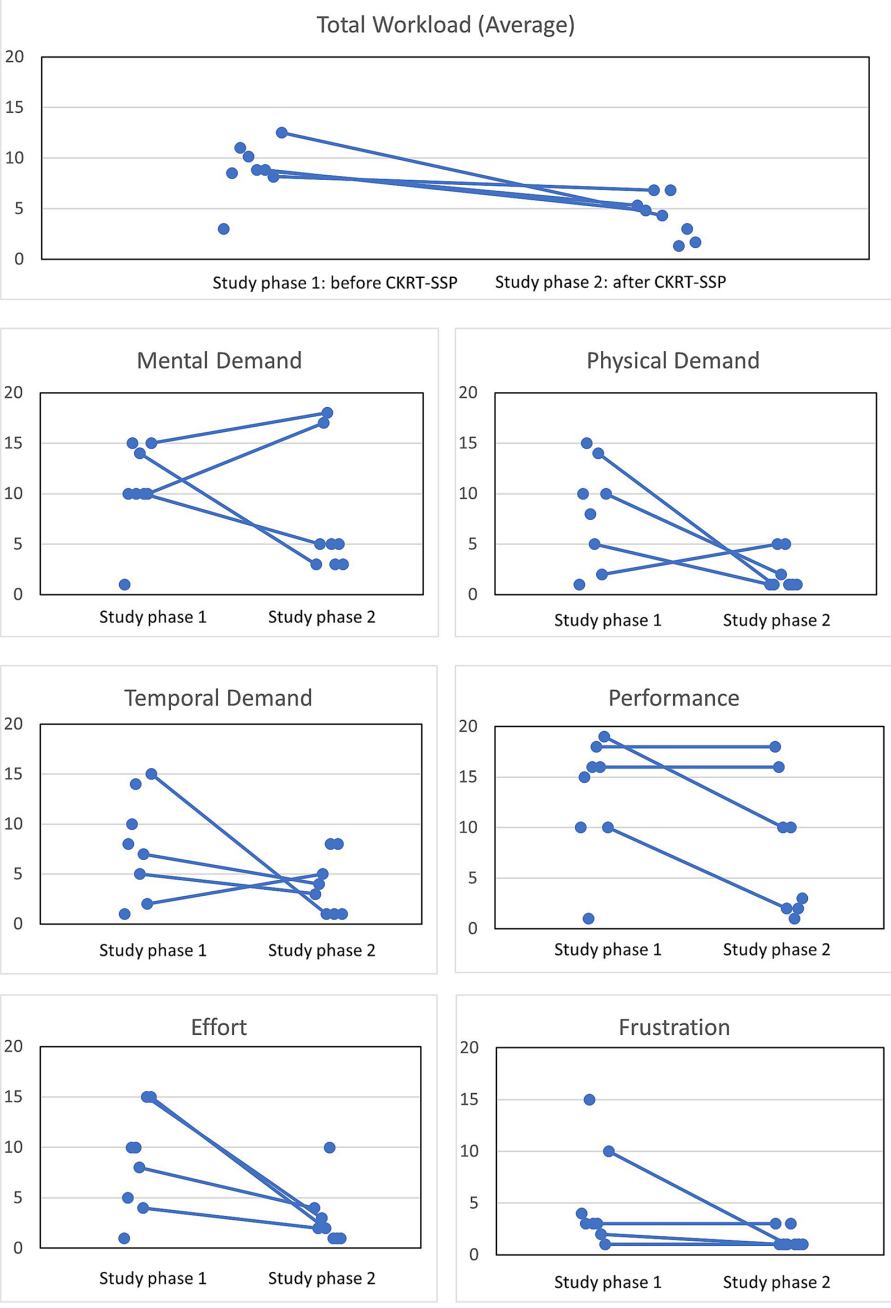
Results of the modified NASA-TLX results from study phase 1 and study phase 2; all results are displayed with a dot in the 6 individual charts representing the subscales, results of the same person are connected by a line, the total workload chart represents the average of the 6 subscales

Figure 5 shows the results modified NASA-TLX from study phase 1 and 2 for the 6 subscales of the modified NASA-TLX as well as the total workload, i.e. the average of the 6 subscales. For the total workload the data suggest a reduction with the CKRT-SSP as there is a reduction for each of the four paired data sets complemented with consistent findings with the unpaired data. This apparently is attributable to mainly the subscales physical demand and effort. With mental demand and performance, we did not identify any sign for an effect, whereas there might be a signal with the subscales temporal demand and frustration.

### Qualitative interview results

Study Phase 1 baseline interviews showed distinct workflows: nurses focused on catheter handling, therapy adjustments, and monitoring; physicians on decision-making, therapy planning, and catheter placement. Key CKRT challenges were filter clotting, alarm fatigue, and anticoagulation issues. Blood gas analysis and lab values were key data points for both groups, nurses additionally cited body weight and temperature. Common device errors involved citrate handling, clamping mistakes, and protocol deviations. Communication was central, with physicians coordinating treatment transitions using whiteboards, and nurses reporting collaboration with pharmacy staff.

Study Phase 2 collected user feedback and identified potential improvement areas during the CKRT-SSP testing and post session interviews. In general, the system was positively commented for user-friendliness, integration within existing IT infrastructure, and retrospective data analysis. Automatic BGA import, visual decision-support tools, and real-time acid-base alerts were valued for enhancing clinical decision-making. Remote monitoring was positively received, but users expressed a desire for more advanced capabilities, including bi-directional communication. Usability issues reported were navigation, data entry, and need for further clarity of patients’ fields, prompting calls for interface improvements. Participants noted the need for clearer role separation, and improved visual alarm prioritization to reduce cognitive load. Smooth integration with hospital systems was also seen as critical for supporting clinical workflows.

## Discussion

### H1:

The results of the SUS demonstrate that the CKRT-SSP usage was perceived as effective, efficient, and satisfying. The answer distribution in Fig. 3 provides a nuanced view of user responses, offering insights into the strengths and areas for improvement of the CKRT-SSP system. Out of 10 questions, only questions 4, 6 and 7 do not have a clear answer distribution, which could be due to various levels of work experience, digital literacy, and the cross-section of the healthcare workforce within the ICU of the test participants. Questions 4 and 6 each had one neutral response but no opposing answers. In contrast, Question 7 showed a wider distribution of responses, including one opposing answer. Overall, the system was perceived as consistent, learnable, and usable without technical assistance. However, one disagreement on Question 7 may suggest variability in the onboarding experience and highlights some early complexity. This may indicate a need for enhanced first-time user support measures, such as introductory tutorials or simplified entry points.

Regarding H2, the UEQ results are overall very positive. The pragmatic quality aspect “clarity”, which evaluates the ease of understanding of a product, achieved the highest value. In the context of the high requirements in the intensive care environment, the result achieved by the prototype suggests that the use of the CKRT-SSP does not entail any “unnecessary” additional cognitive workload.

Attractiveness, a valence dimension, reflecting an emotional reaction on an acceptance/rejection dimension [17], reached the second highest result. Introducing a new or even an additional product in an environment such as the intensive care unit may already be met with rejection since it is new or additional. This high value of the CKRT-SSP is promising for a product designed in this way regarding future user acceptance in clinical use.

Compared to the other categories, the hedonic quality aspect “novelty” achieved the lowest value but has still a neutral to positive rating. Hedonic quality rather describes aspects that relate to the pleasure or enjoyment of using a product and do not relate directly to tasks and objectives [17]. Regarding the UEQ categories relevant for the prototype, the hedonic quality aspects play a subordinate role compared to the pragmatic ones. Although the aim is to give the user a positive feeling while carrying out their tasks using a product, the direct support for tasks and user acceptance are more important in the context of intensive care medicine. Nevertheless, these aspects are known to influence motivation and long-term user engagement [16]. The relatively lower scores in novelty may reflect the prototype’s current emphasis on core functionality and highlight areas for further development to enhance engagement and long-term acceptance in clinical settings.

The usability and user experience results observed for the CKRT-CDSS prototype (SUS median 87.5; UEQ > 0.8 across most dimensions) are comparable to those reported for a similar ICU-specific tool [20]. According to established benchmarks, SUS scores above 85 indicate excellent usability [15]. High scores in the UEQ dimensions clarity and dependability reflect that participants perceived the system as easy to understand and reliable, which are known factors contributing to CDSS acceptance in critical care environments [21, 22].

Regarding H3 and according to the available results, total workload has consistently been reported lower by all four persons participating in both study phases which is consistently complemented by the results of the remaining participants. Thus, we consider the reported results supporting H3, i.e. a total workload reduction with the CKRT-SSP. An explicit confirmation, however, would require further data. The two components physical demand and effort of the modified NASA-TLX apparently contribute substantially to this finding. The low rating of physical demand when using the CKRT-SSP might have resulted from an unconscious comparison with actions on the CKRT device, as operating tasks on the device (e.g., changing bags) is physically more demanding than the operation of software such as the CKRT-SSP (e.g., mouse clicks). It should be noted that the physical requirements and actions on CKRT device will not change as such with the introduction of CKRT-SSP, however it can be anticipated that overall physical strain might be reduced. This is because remote access has positive effects, as walking routes for interacting with the device can be planned more specifically and can be better integrated into the overall work process. For future studies, the question targeted to assess physical demand should be more clearly specified in the context of software interaction to improve clarity and interpretability of workload assessments. In addition, domain-specific modifications of NASA-TLX such as the SURG-TLX have been established [23] and NASA-TLX analogously could be adapted to better reflect ICU-specific software tasks. The use of the CKRT-SSP also had a significant influence towards less perceived effort, which can possibly be explained by the clarity of the prototype and the efficient achievement of goals when solving the work tasks (from the test scenario). In addition, it can be concluded that operations of the CKRT device (preparing, set-up, priming, and patient connecting) involves numerous and diverse tasks, which may inherently lead to a perception of more effort and frustration compared with the use of CKRT-SSP. Nevertheless, the results show that there is a trend towards a positive impact and underline that the CKRT-SSP has the potential to reduce workload in some of the six subscales. The observed reduction in workload in our study is consistent with previous findings that well-designed CDSS tools can decrease the cognitive and physical demands on ICU staff [24]. The aspect of reduction in workload is of relevance given an existing high burnout rate among nurses [25], which needs considerable counteractions.

Qualitative insights indicate that some challenges reported in the current CKRT practice (e.g., filter clotting, alarm fatigue) can potentially be addressed by functionalities of the CKRT-SSP. For example: a) automatic BGA import, and real-time acid-base alerts could support anticoagulation management; b) real-time alerts could address alarm fatigue by prioritizing critical warnings c) visual decision-support tools may assist therapy adjustments and clinical decisions; d) remote monitoring could ease data access, although also concerns about remote control were noted; and e) integration with PDMS/EMR systems and retrospective analysis could support learning from past treatment errors such as protocol deviations.

Our study has several limitations. First, although the study was based in the ICU, the tests themselves were simulated and did not involve real patients and treatments; therefore, they cannot fully represent the real working environment. Secondly, the potential for reference bias among respondents. Respondents may have unconsciously compared actions required for CKRT-SSP with the actions on a CKRT device. It should be noted that the physical requirements and actions on a CKRT device will not change as such with the introduction of CKRT-SSP, but rather that the standard practice (i.e., overall workflow in the ICU when treating AKI patients) could be positively influenced. Thirdly, the sample size is small with a total of 12 participants in a single center only, which limits the generalizability of our conclusions. In addition, the only partial overlap of study participants in study phases 1 and 2 limited the possibilities of comparative analysis, e.g., paired statistical tests could not be used. Such limited number of study participants was due to the restricted availability of healthcare professionals at the study site but is still matches established practices in early-stage usability testing [26, 27].

Further there are conceptual limitations with CDSS. It has been suggested that implementation of CDSS can raise ethical concerns due to algorithmic bias with inequal treatment outcomes, legal issues in terms of data privacy, and lack of clinician acceptance [11]. Earlier studies have also highlighted common barriers to adoption, including poor integration into workflows, alert fatigue, and limited clinician trust [28]. Additionally, in the ICU environment, patients often have multiple comorbidities, which may make integration of multiple systems for multiple disease entities challenging. The UX study did not evaluate these aspects, and further research is needed to assess the impact of CKRT-SSP during clinical application. Such further research should entail: a) conducting a study in real ICU settings to assess the impact on the work environment, learning curve, and effort required for implementation into routine workflows, including integration with existing clinical information systems and other decision support tools; and b) assessment of the long-term impact of CKRT-SSP on workload, cognitive load and c) evaluating CKRT-SSP across diverse ICU environments with different and larger user groups to support the generalizability.

## Conclusion

This prototype offers promising indications regarding its attractiveness, applicability, and usability, which speak in favor of developing such a system. The ease of use (effectiveness and efficiency) and a comprehensible presentation of the relevant data points (clarity) with this CDSS prototype appear to have been key for the overall positive perception by the interviewed HCP. The results of this study have shown that CKRT-SSP has the potential to reduce workload and possibly even mitigate cognitive overload contributing to the understanding whether and how clinical decision support systems (CDSS) impact cognitive workload. We obtained valuable insights for the further development of this CKRT-SSP with the goal to improve the work environment of HCPs and thereby to indirectly improve patient care. This is an advantage of the UX testing during the development phase, since the findings that emerge directly during test use can be incorporated into further development even before the software enters the market [29]. Further research is needed to test the software under real conditions in the busy ICU environment and confirm the accuracy of the results.

## Electronic supplementary material

Below is the link to the electronic supplementary material.

Supplementary Material 1

Supplementary Material 2

Supplementary Material 3

## Data Availability

The data that support the findings of this study are available from the corresponding author, L.-M. Kunz, upon reasonable request.
