# Supplementary material for: User experience study to evaluate a clinical decision support system prototype supporting continuous kidney replacement therapy in a simulated ICU environment
Source: BMC Med Inform Decis Mak. 2025 Sep 10;25:328. doi: 10.1186/s12911-025-03165-7 (PMC12424209; doi:10.1186/s12911-025-03165-7)
Supplement: Supplementary file 1 — Supplementary Material 1 [file 12911_2025_3165_MOESM1_ESM.pdf]

FRESENIUS
MEDICAL CARE

Study Phase 1
Guiding Questions Baseline Interviews

1) Please describe in general terms your activities or workflow about the acute dialysis of your ICU patients.

2a) (ICU Overview) How to obtain an overview of patient statuses and ongoing therapeutic interventions.
at the start of the shift:
during operation:
How much time do you spend on this?
How much mental effort is required?
Where are potential errors?
Wishes/Comments:

2b) (Prescription) How to obtain information on indications for planned dialysis.
at the start of the shift:
during operation:
How much time do you spend on this?
How much mental effort is involved?
Where are potential errors?
Wishes/Comments:

2c) (Monitoring) How to obtain an overview of ongoing dialysis therapies.
at the start of the shift:
during operation:
How much time do you spend on this?
How much mental effort is involved?
Where are potential errors?
Wishes/Comments:

2d) (Treatment End) How do you get the information for ending dialysis therapy / that at the start of a shift:
during operation:
How much time do you spend on this?
How much mental effort is required?
Where are potential errors?
Wishes/Comments:

3) What are the biggest challenges regarding dialysis treatments?

4) What data is important to you in dialysis and how is it used?

5) Do you include patient weight/patient size in the dialysis prescription?
If Yes: When and How

6) What typical "mistakes" happen during dialysis treatments? How do you deal with these errors (error feedback, discussion, etc.)?

7) With whom do you communicate regarding dialysis treatment and which topics are discussed?

8) Please explain how other treatments on the patient affect dialysis and how you deal with them.

9)Please explain if and how you statistically evaluate your dialysis treatments. (Benchmarking)
How much time do you spend on this?
How much mental effort is required?
Where are potential errors?
Wishes/Comments:

General questions:

10) What things/features/functions about medical equipment other than dialysis machines do you value most?

11) What things/features/functions about medical devices other than dialysis machines do you tend to see as negative?

Feature	Without Usage	With Usage
ICU Overview	How do users today get an overview of ongoing treatments<br>What is the required time<br>How is the workload<br><br>How do users create a new CKRT prescription today<br>What is the required time<br>How is the workload<br><br>How do users know if CKRT therapy is running smoothly?<br>What is the required time<br>How is the workload	
Patient specific CKRT therapy prescription	How does the user get all relevant data like patient weight for the CKRT prescription<br>What is the required time<br>How is the workload<br><br>How does the user get all relevant data like BGA data for the CKRT prescription<br>What is the required time<br>How is the workload<br><br>How does the user prescribe CKRT<br>What is the required time<br>How is the workload<br><br>How does he incorporate factors such as renal dose when prescribing<br>What is the required time<br>How is the workload<br><br>How does the user include the acid bases graph when prescribing<br>What is the required time<br>How is the workload	
Patient specific CKRT therapy monitoring	How does the user get a full therapy overview of the treatment in progress<br>What is the required time<br>How is the workload<br><br>How does the user get all relevant data like BGA data for the CKRT monitoring<br>What is the required time<br>How is the workload<br><br>How does he incorporate factors such as renal dose while monitoring the therapy<br>What is the required time	

	How is the workload<br><br>How does the user include the acid bases graph while monitoring the therapy<br>What is the required time<br>How is the workload<br><br>How does the user the ultrafiltration monitore<br>What is the required time<br>How is the workload<br><br>How is the balancing documented<br>What is the required time<br>How is the workload<br><br>How are anticoagulation settings adjusted and what information is required to do so (heparin vs. CiCa)<br>What is the required time<br>How is the workload<br><br>How do handovers to the following shift take place regarding the CKRT therapy and the device?<br>What is the required time<br>How is the workload
Benchmarking / quality metrics	How does the user evaluate the quality of treatments in the ICU?<br>What is the required time<br>How is the workload
