# Supplementary material for: User experience study to evaluate a clinical decision support system prototype supporting continuous kidney replacement therapy in a simulated ICU environment
Source: BMC Med Inform Decis Mak. 2025 Sep 10;25:328. doi: 10.1186/s12911-025-03165-7 (PMC12424209; doi:10.1186/s12911-025-03165-7)
Supplement: Supplementary file 2 — Supplementary Material 2 [file 12911_2025_3165_MOESM2_ESM.pdf]

FRESENIUS MEDICAL CARE

Study Phase 2
CKRT-SSP Use Scenarios for UX Testing

	USE SCENARIO A	USE SCENARIO B1	USE SCENARIO B2	USE SENARIO C
Core Task	**A ICU Overview / CKRT therapy Dashboard**	**B1 Patient specific CKRT therapy prescription**	**B2 Patient specific CKRT therapy monitoring**	**C Benchmark the treatments**
Sub tasks	A1 Perform access to the system "CKRT-SSP"	B1.1 Control and adjust the patient data	B2.1 Perform check of Patient Data	C1.1 Control and adjust the time span
	A2 perform check of running treatments	B1.2 Perform check of BGA values	B2.2 Perform check of respective dialysis parameter	C1.2 Perform check of "effective treatment time"
	A3 perform check of connectivity status	B1.3 Control and adjust the treatment mode	B2.3 Monitor the pressure curves	B1.3 Perform check of "CKRT treatment time per patient"
	A4 perform check of treatment duration	B1.4 Control and adjust the respective dialysis parameter	B2.4 Monitor the BGA data	C1.4 Perform check of "treatment time lost"
	A5 perform check of renal dose	B1.5 Control and adjust the respective anticoagulation setting	A2.5 Monitor the current renal dosage	C1.5 Perform check of "filter life"
	A6 respond to indications regarding running treatment	B1.6 Control and adjust the respective renal dosage	B2.6 Monitor the accumulated UF	C1.6 Perform check of "patients with CKRT treatment"
	A7 select the bed location of the desired patient to receive treatment	B1.7 Control and adjust the respective UF goal	B2.7 Control and adjust the respective dialysis parameter	C1.7 Perform check of "delivered dose"
	A8 select the bed location of the desired patient to access running treatment	B1.8 select the desired disposable for the treatment	B2.8 Control and adjust the anticoagulation	
		B1.9 Perform check of treatment prescription	B1.9 Control and adjust the acid base correction	
			B2.10 Perform check of therapy history (e.g. balance data, balance history, events)	

1)
