# Supplementary material for: User experience study to evaluate a clinical decision support system prototype supporting continuous kidney replacement therapy in a simulated ICU environment
Source: BMC Med Inform Decis Mak. 2025 Sep 10;25:328. doi: 10.1186/s12911-025-03165-7 (PMC12424209; doi:10.1186/s12911-025-03165-7)
Supplement: Supplementary file 3 — Supplementary Material 3 [file 12911_2025_3165_MOESM3_ESM.pdf]

FRESENIUS
MEDICAL CARE

Study Phase 2
Guiding Questions Post-Session Interviews

1) Please describe in general the experience you had with the CKRT-SSP in the test.
   What did you notice positively/negatively?"
2) Did you feel that you had made mistakes?
3) Please evaluate the process of prescribing therapy.
   What was positive/negative.
   Was all the data/details available that you need?"
4) Please evaluate the therapy monitoring.
   What was positive/negative.
   Was all the data/details available that you need?"
5) Please rate the dialysis statistics in the CKRT-SSP.
   What was positive/negative.
   Was all the data/details available that you need?"
6) Do you have any other comments?
7) Would the CKRT-SSP help/simplify your workflow?
   If yes why
   If no why
8) Prescription UF-Goal so meaningful? Do you use the UF-Goal according to the concept as implemented in the CKRT-SSP?
   If not, what are the differences?
9) If dialysis is continued after an outlet attempt: Would you then continue dialysis with the previous parameters?

Further Questions

1) How are patient data and CKRT treatment data currently documented in your department?
   Are you satisfied with the current CKRT data documentation in your department?
2) Are you satisfied with the accessibility/ visibility of CKRT data documented in your department?
3) How much time do you spend actively looking/ searching for the patient and treatment data for decision-making?
4) Would a clear graphical representation be beneficial in your department? If yes in which way? Probe: Improved workflow, reduced workload.
5) Would you benefit from software to help you prescribe CKRT and then track remotely the dialysis dose effectively delivered?
6) How and according to which criteria do you currently determine the dialysis dose to be prescribed?
7) How important is a guideline compliant CKRT dose to you?
8) Would software support help you to achieve this?
9) How important would this be for you?
10) Would closer alignment of prescribed vs delivered dose positively impact treatment standardization in your department?
11) Would you like to be able to track CKRT data remotely (independent of being in the patient's room) and what value would that bring to you?

Probe: Improved workflow, easier handoff between shift changes, more predictable patient visits, reduced workload

12) Would you find it labor-saving to have insight into and influence CKRT treatment data anywhere and anytime?
13) Are retrospective treatment/patient/ machine data currently being analyzed in your department? If yes, how? - Would software support with benchmarking/ quality metrics bring value to your department? Probe for: comparison with internal clinical SOPs, optimal planning by identifying potential treatment inefficacies and related cost drivers (e.g., filters, dialysate bags, technical performance of machines etc.)
14) Do you see that the CKRT-SSP. can be a cost saving tool in your department and in which way? Probe: Reduced inefficiencies via Blackmarking and Quality metrics, improved workflow "
